# DAILY DYNAMICS AMONG AFFECT, PHYSICAL ACTIVITY, AND SLEEP: A MULTILEVEL AUTOREGRESSIVE CROSS-LAGGED MODEL

**DOI:** 10.1093/geroni/igae098.2704

**Published:** 2024-12-31

**Authors:** Sun Ah Lee, Zachary Fisher, David Almeida

**Affiliations:** The Pennsylvania State University, University Park, Pennsylvania, United States; The Pennsylvania State University, University Park, Pennsylvania, United States; Pennsylvania State University, University Park, Pennsylvania, United States

## Abstract

Previous research suggests that physical activity and sleep are important behavioral factors contributing to affective well-being. However, less is known about their simultaneous dynamics in daily life. The current study examines the within-day and across-day associations among affect, moderate-to-vigorous physical activity (MVPA), and sleep. Data were drawn from the third wave of a daily diary study of Midlife in the United States (MIDUS). A sample of 1,171 middle-aged and older adults (Mage= 62.61; SDage = 10.26: 57% female; 82% white) completed 9,059 daily telephone interviews across eight consecutive evenings, where they reported daily positive (PA) and negative affect (NA), previous-night sleep duration and sleep quality, and MVPA engagement. Multilevel autoregressive cross-lagged models were estimated to simultaneously address the bidirectional relationships among daily affect, MVPA, and sleep. For within-day correlations, MVPA engagement was correlated with same-day higher PA and lower NA. Longer sleep duration was correlated with the same-day higher sleep quality. For across-day associations, longer sleep duration and higher sleep quality predicted higher PA and lower NA on the next day. Higher sleep quality also predicted a greater likelihood of next-day MVPA. In addition, higher PA predicted higher sleep quality and a greater likelihood of MVPA on the following day. However, previous-day MVPA predicted higher next-day NA. Findings highlight the benefits of sleep on daily affective well-being and MVPA engagement, as well as the importance of positive affect on MVPA and sleep. This suggests that managing adequate sleep and emotions could be potential targets for prevention/intervention in promoting healthy aging.

